# Assessing scale‐wise similarity of curves with a thick pen: As illustrated through comparisons of spectral irradiance

**DOI:** 10.1002/ece3.4496

**Published:** 2018-09-12

**Authors:** Saara M. Hartikainen, Agnieszka Jach, Aurea Grané, Thomas Matthew Robson

**Affiliations:** ^1^ OEB, Viikki Plant Science Centre University of Helsinki Helsinki Finland; ^2^ Department of Finance and Statistics Hanken School of Economics Helsinki Finland; ^3^ Department of Statistics Universidad Carlos III de Madrid Getafe Spain

**Keywords:** canopy, cross‐dependence, multi‐scale, phenology, spectral irradiance, spectroradiometer, sunfleck, thick pen measure of association, thick pen transform, understorey

## Abstract

Forest canopies create dynamic light environments in their understorey, where spectral composition changes among patterns of shade and sunflecks, and through the seasons with canopy phenology and sun angle. Plants use spectral composition as a cue to adjust their growth strategy for optimal resource use. Quantifying the ever‐changing nature of the understorey light environment is technically challenging with respect to data collection. Thus, to capture the simultaneous variation occurring in multiple regions of the solar spectrum, we recorded spectral irradiance from forest understoreys over the wavelength range 300–800 nm using an array spectroradiometer. It is also methodologically challenging to analyze solar spectra because of their multi‐scale nature and multivariate lay‐out. To compare spectra, we therefore used a novel method termed thick pen transform (TPT), which is simple and visually interpretable. This enabled us to show that sunlight position in the forest understorey (i.e., shade, semi‐shade, or sunfleck) was the most important factor in determining shape similarity of spectral irradiance. Likewise, the contributions of stand identity and time of year could be distinguished. Spectra from sunflecks were consistently the most similar, irrespective of differences in global irradiance. On average, the degree of cross‐dependence increased with increasing scale, sometimes shifting from negative (dissimilar) to positive (similar) values. We conclude that the interplay of sunlight position, stand identity, and date cannot be ignored when quantifying and comparing spectral composition in forest understoreys. Technological advances mean that array spectroradiometers, which can record spectra contiguously over very short time intervals, are being widely adopted, not only to measure irradiance under pollution, clouds, atmospheric changes, and in biological systems, but also spectral changes at small scales in the photonics industry. We consider that TPT is an applicable method for spectral analysis in any field and can be a useful tool to analyze large datasets in general.

## 1 INTRODUCTION

A measurement of spectral irradiance can be viewed as a curve made up of rough and smooth components: The rough parts are linked with variations in the spectrum occurring over small scales, while the smooth parts are linked with large‐scale variations (scale is defined as the number of units of the *x*‐variable, here wavelength, hence scale equals the number of nm). In this paper, we present a novel approach for multi‐scale comparison of curves recorded under different experimental conditions.

The incident solar irradiance at ground level is influenced by a number of atmospheric and environmental factors, which determine the relative contribution of direct and diffuse radiation to the global (direct + diffuse) solar radiation. Furthermore, different regions of the solar spectrum are differentially affected due to selective absorption, transmittance, and reflectance by, for example, atmospheric gases, particles, or organic material. In addition to these fundamental considerations, the time of year and nearby physical structures both interact with radiation and can significantly alter the incident irradiance at a given location (Federer & Tanner, 1966; Grace, 1983; Smith, 1982). One such factor is the position under a forest canopy (Coombe, 1957; Dengel, Grace, & MacArthur, 2015; Freyman, 1968; Hutchison & Matt, 1977; Urban et al., 2007; Vezina & Boulter, 1966). Transitions between shade and sunflecks involve changes in the irradiance received by plants and its spectral composition, where they mediate physiological responses to optimize exploitation of brief favorable light conditions (Campany, Tjoelker, von Caemmerer, & Duursma, 2016; Chen, Zhang, Li, & Cao, 2011).

Technological advances in the manufacture of small portable spectroradiometers, that use linear array detectors to simultaneously measure a spectrum, allow multiple successive snapshots of highly dynamic environments such as forest understoreys to be captured within seconds or even faster. This contrasts with a scanning spectroradiometer which would scan across the wavelength range over a period of time taking up to minutes to record a single solar spectrum. Despite the inferior optical performance of array spectroradiometers compared to scanning spectroradiometers, they can accurately capture the micro‐environmental variability at high resolution if used judiciously (Björn et al., 2012; Nevas, Teuber, Sperling, & Lindemann, 2012; Seckmeyer et al., 2010).

A particularly interesting research question is how the stand structure and identity of canopy tree species influence the properties of the incident spectral irradiance at the forest floor. Past studies suggest that some differences in spectral composition may occur among deciduous trees (Federer & Tanner, 1966; Messier & Belleeur, 1988). However, as a result of the technical restrictions described earlier, it has proved difficult to distinguish the effects of seasonal variation (Baldocchi, Hutchison, Detlef, & McMillen, 1984; Floyd, Burley, & Noble, 1978) from those of sunlight position (Vezina & Boulter, 1966 and references therein, and Leuchner, Hertel, & Menzel, 2011), as influenced by stand architecture (Parker, Davis, & Chapotin, 2002; Stoutjesdijk, 1972; Yang, Miller, & Montgomery, 1993) and optical properties of leaves (Knapp & Carter, 1998; Messier & Belleeur, 1988). Since the light environment in the understorey is so dynamic, an instantaneous spectrum captured with an array spectroradiometer may yield more useful information than a scanning spectroradiometer. Such a spectrum also lends itself to analysis with a method that considers the whole range of wavelengths captured, to extract as much information as possible from the data.

Addressing the challenge of how to extract multi‐scale information from these spectra requires innovative approaches to the analysis of irradiance. We apply a methodology of Fryzlewicz and Oh (2011), which has never been used in ecological context, to identify differences among instantaneous spectra from forest understoreys related to canopy type, season, and sunlight position, as part of an extensive study surveying solar irradiance in forest understoreys.

Typical measures of spectral composition are integrals and ratios (of these integrals) of spectral irradiance: for example, photosynthetically active radiation, PAR, which is obtained by integrating spectral irradiance from 400 to 700 nm and the red to far‐red ratio, R:FR, given by dividing the integrated spectral irradiance over [655, 665] nm and over [725, 735] nm (Smith, 1982). In contrast, we take into account entire spectra, for all wavelengths from 300 to 800 nm rather than the integrals. We also consider the multi‐scale nature of the spectral irradiance by carrying out the analysis with respect to both its rough features (associated with variations over bands of several nm) and its smooth features (associated with variations over bands of tens of nm).

This novel analysis is facilitated by thick pen transform (TPT) of Fryzlewicz and Oh (2011), which boils down to drawing (measurements of) spectral irradiance with pens of different thickness: Small thickness is linked with rough features and large thickness with smooth features. Other benefits of our approach include that the thick pen transform allows us to quantify cross‐dependence or similarity between two or more spectra (thickness‐wise) via thick pen measure of association (TPMA), which is also visually interpretable. We also stress the fact that the comparisons can be made for more than two spectra, which is in contrast to, for example, a correlation coefficient. Lastly, this methodology is applicable to non‐equispaced measurements, hence no interpolation is needed.

We demonstrate the utility of this approach in addressing the following questions related to spectral irradiance in forest understoreys: (a) How is the spectrum modified by the time of year during spring canopy leaf‐out? (b) Do stands comprising different canopy types (structure and tree species) affect the spectrum differently? (c) To what extent is the solar irradiance spectrally different at distinct sunlight positions (sunfleck, semi‐shade, and shade) in the understorey of a forest stand? (d) Are the impacts of sunlight position, stand, and time of year on the rough part of the spectrum different from the impacts of these factors on the baseline part of the spectrum?

## 2 MATERIALS AND METHODS

### 2.1 Description of the study

Measurements were carried in forest stands adjacent to Lammi Biological Station, southern Finland (61°3.24′N, 25°2.23′E) during spring, 2015. The spectral irradiance was measured in five different stands, chosen based on their age and canopy composition: Two *Betula* sp. L. stands differing in age, plus one of *Betula* sp. L. mixed with other broad‐leaved species, and a pure stand of *Quercus robur* L. and *Picea abies* (L.) H. Karst. We refer to the stands as “BetulaOld,” “BetulaYoung,” “BetulaOldMixed,” “Quercus,” and “Picea,” respectively. All stands were large enough to create their own understorey light environment with minimal interference from their surroundings.

Four independent measurement points were chosen that were typical of each stand, located approximately equidistant between the nearest canopy trees to assure comparability of replicates and to avoid obvious variability in irradiance related to proximity of trunks. To exploit the capability of the array spectroradiometer for instantaneous measurements, we made a distinction between three categories of understorey position with respect to sunlight (henceforth “position”) recorded at each measurement point: “shade,” “semi‐shade,” and “sunfleck.” Sunflecks were measured from a position receiving predominately direct radiation passing through the canopy between small gaps in the crown with most of the diffuse radiation intercepted by the surrounding canopy after leaf‐out, defined by Smith and Berry (2013) as receiving less than full solar irradiance and for a duration shorter than 8 min, or measured prior to leaf‐out as larger sunpatches. Conversely, the shade position was measured from within the umbra (shadow) of a tree trunk receiving entirely diffuse radiation, while in the semi‐shade position, radiation was assessed visually as being transmitted through leaves of the upper canopy, producing irradiance in between sunflecks and shade.

Data were also collected from a nearby open field area at the beginning, in the middle, and at the end of the daily sampling period to control for factors that can potentially affect the solar spectrum such as weather (e.g., atmospheric water vapor or the ozone column thickness) and time from solar noon. We labeled these recordings as “OpenBeg,” “OpenCen,” “OpenEnd,” respectively. When compared to other canopy positions, we refer to them as “full‐sun.”

To minimize the impact of the time of day and control for the other variables listed, we conducted the measurements at all sites on the same day on three occasions, in April, May, and June, and as close as possible to solar noon. The measurements were taken from exactly the same marked places in the understorey on each date: 25 April 2015, 22 May 2015, and 05 June 2015. These dates were chosen to allow us to follow changes with the advancing spring phenology of the canopy and understorey species, and to coincide with the clearest possible weather condition during that period.

By definition, following leaf phenology implies that leaf‐out must have started before a leaf semi‐shade measurement can be taken. For this reason, during early leaf‐out, semi‐shade was not present in all stands on 25 April 2015, nor on 22 May 2015 in the Quercus stand, because *Q. robur* started to leaf out later than *Betula*.

Apart from measurements of spectral irradiance, the basal area (per m^2^/ha) and density of trees (per ha) in each stand were recorded at the time of the first measurements in April 2015 (Table 1). The plant area index (PAI, per m^2^/m^2^) at each measurement point was estimated from hemispherical photographs in overcast weather to ensure a homogeneous sky providing good contrast with the canopy. Photographs were taken using a Nikon D7100 camera body (Nikon Corporation, Tokyo, Japan) with a sigma 4.5 mm F2.8 EX DC HSM circular fisheye lens (Sigma Corporation of America, Ronkonkoma, NY) in RAW format. The camera was set on a tripod with a leveling base at 40 cm height from the ground. Several photographs with the same aperture f 20/22 and ISO 200, but with different exposure times, were acquired from each measurement point per date. Exposure time was determined manually through visual inspection, with the shortest exposure maximizing potential gaps and the longest exposure excluding any overexposure of the upper canopy, to obtain the correct range of PAI. Pre‐processing of photographs was done following an updated protocol of Macfarlane, Ryu, Ogden, and Sonnentag (2014) (C. Macfarlane, personal communication). To reduce variation related to exposure time, binarization of the pre‐processed images was also made by using the Floyd–Steinberg dithering option in IrfanView 4.44 (Irfan Skiljan, Wiener Neustadt, Austria), and using the softwareʼs standard binarization algorithm. All versions of the original photographs were analyzed in Hemisfer 2.16 (Patrick Schleppi, WSL, Switzerland) applying the automatic threshold algorithm (Nobis & Hunziker, 2005) for PAI. The equation from Miller (1967) was used to resolve the gap‐fraction‐inversion model, and foliage clumping was considered according to Chen and Cihlar (1995), combined with a non‐linearity and slope correction method (Schleppi, Conedera, Sedivy, & Thimonier, 2007).

**Table 1:** Stand characteristics (mean ± standard error from four measurement points)

	BetulaOld	BetulaOldMixed	BetulaYoung	Picea	Quercus
Tree basal area	34.3 ± 1.70	23.0 ± 1.80	21.8 ± 1.10	39.0 ± 2.30	21.5 ± 0.60
Tree density	1,200 ± 115.5	900 ± 100.0	1,550 ± 150.0	750 ± 95.7	400 ± 141.4
PAI 2015‐04‐25	1.1 ± 0.10	1.3 ± 0.10	1.2 ± 0.04	3.9 ± 0.10	1.1 ± 0.20
PAI 2015‐05‐22	1.4 ± 0.02	2.2 ± 0.10	2.6 ± 0.10	4.0 ± 0.10	1.2 ± 0.03
PAI 2015‐06‐05	2.4 ± 0.10	3.5 ± 0.05	3.6 ± 0.20	4.0 ± 0.10	2.7 ± 0.10

*Notes*

Tree basal area (per m^2^/ha) and density (per ha) were recorded in April 2015, and plant area index (PAI, per m^2^/m^2^) was estimated from hemispherical photographs corresponding to each measurement date.

### 2.2 Measurement of spectral irradiance

Spectral (energy) irradiance (W m^−2^ nm^−1^) was measured with a CCD array spectroradiometer Maya 2000 Pro (Ocean Optics, Dunedin, FL, USA) attached via a fiber‐optic cable (FC‐UV400‐2 400‐μm, Avantes, Leatherhead, UK) to a D7‐H‐SMA cosine diffuser (Bentham Instruments Ltd., Reading, UK) with spectral range of 200–1,100 nm. The spectroradiometer was calibrated by the Finnish Radiation and Nuclear Safety Authority (STUK) (Ylianttila, Visuri, Huurto, & Jokela, 2005) prior to the measurements in early spring, to allow accurate recording of outdoor solar radiation from the UV‐B to the near‐infrared. A further stray‐light correction for the UV and “dark noise” correction were applied to each recording of solar spectral irradiance, through its repetition with a polycarbonate cap over the diffuser blocking solar UV radiation (UV‐B 280–315 nm plus UV‐A 315–400 nm) and a dark cap over the diffuser blocking UV and visible solar radiation. Pre‐processing of the data was performed using the routine process_maya_files from R package MayaCalc in R (R Core Team 2015).

Sets of up to 100 contiguous measurements of spectral irradiance were recorded with the diffuser exactly horizontal held on a tripod 40 cm from the ground. The integration time for each set of spectra was adjusted manually to give the maximum precision. This was assessed through the number of counts of pixels registered on the array. Spectra were screened out of this set when at any given wavelength the maximum sensitivity of the array was exceeded or fell under the minimum threshold required for accuracy in the UV region (Aphalo, 2016).

The spectral irradiance was recorded for the following non‐equispaced grid of wavelengths 250.14, 250.62, 251.09, 251.57, … , 899.77 nm. The spectra presented and compared in these analyses are restricted to [300, 800] nm. These limits were determined by the region of calibration of the spectroradiometer but also for their biological significance because known plant photoreceptors are active within this region of the spectrum. Because of our requirement for high confidence in the data, we set the minimum wavelength in the UV‐B at 300 nm excluding the very small portion of spectral irradiance <300 nm from the analyses because of reduced precision of some measurements in this range. The wavelength interval [300, 800] nm was discretized via 1,091 points, with the minimum gap between two consecutive points of 0.44 nm and the maximum of 0.48 nm.

**Figure 1:**
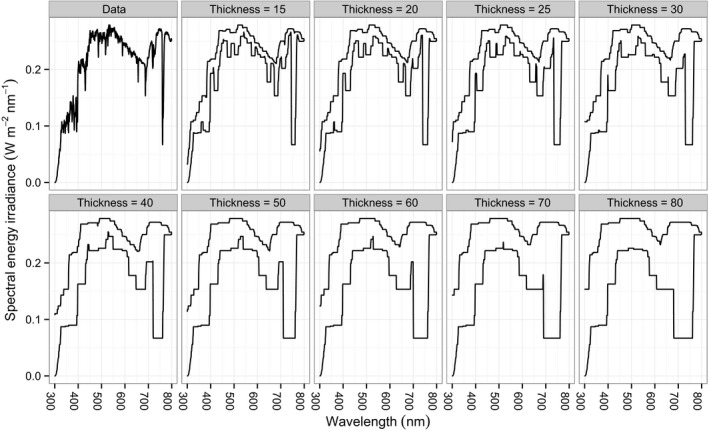
Spectral energy irradiance (W m^−2^ nm^−1^) plotted with a line and with pens of varying thickness. Data collected on the 22 May 2015 around solar noon in the BetulaOld stand in semi‐shade

Every set of up to 100 contiguous measurements, covering a period of <1 s in sunflecks to <20 s in shade, was averaged at each measurement point. The average over the four measurement points from each stand for each position provided the input data, an example of which is shown in the top‐left panel of Figure 1.

We denote such input data by $X={\left\{X_{\lambda }\right\}}_{\lambda \in [\lambda_{\min},\lambda_{\max}]}$, where $\lambda_{\min}=300$ and $\lambda_{\max}=800$. From now on, we will refer to *X* as “a measurement of spectral irradiance” or “spectral irradiance” for short.

### 2.3 Thick pen transform and thick pen measure of association

Let *X* be the input record of the spectral irradiance. Consider a set of *n* thickness parameters (in nm) $T=\{\tau_{1},\tau_{2},\ldots ,\tau_{n}\}$, which are positive constants. For all λs and all $\tau_{i}$s define the following quantities

(1) $$L_{\lambda }^{\tau_{i}}\left(X\right)=\min_{\lambda \leq l\leq \lambda +\tau_{i}}X_{l},\;U_{\lambda }^{\tau_{i}}\left(X\right)=\max_{\lambda \leq l\leq \lambda +\tau_{i}}X_{l},$$

which represent the lower and upper boundaries of the area covered by a square pen, respectively. The result of plotting the data with pens of varying thickness is shown in Figure 1 (the pairs of black lines correspond to $L_{\lambda }^{\tau_{i}}\left(X\right)$ and $U_{\lambda }^{\tau_{i}}\left(X\right)$).

A thickness parameter smaller than the shortest sampling interval, here 0.44 nm, leads to $L_{\lambda }^{\tau_{i}}\left(X\right)=U_{\lambda }^{\tau_{i}}\left(X\right)=X_{\lambda }$; hence, in our study, we take $\tau_{i}\geq 0.44$ nm. The thick pen transform of *X* is a collection of *n* pairs of boundaries denoted as

(2) $${\text{TP}}_{T}\left(X\right)={\left\{{\left(L_{\lambda }^{\tau_{i}}\left(X\right),U_{\lambda }^{\tau_{i}}\left(X\right)\right)}_{\lambda \in [\lambda_{\min},\lambda_{\max}]}\right\}}_{i=1}^{n}.$$

If we assemble all the boundaries for all thickness parameters from Figure 1, then we will obtain the corresponding thick pen transform. The choice of thickness values lies with the analyst and is linked with the particular task at hand. In this paper, we select a set of $\tau_{i}$s ranging from small (15 nm) to large (80 nm), because we are interested in multi‐scale comparisons of spectral irradiance. Accordingly, transformation with a thickness of 15 in Figure 1 outlines fine features of the spectral irradiance and results in a ragged graph, while transformation with a thickness of 80 brings out trend‐like features of the spectral irradiance, resulting in a smooth graph while allowing us to remain ignorant about the small‐scale content of the data.

**Figure 2:**
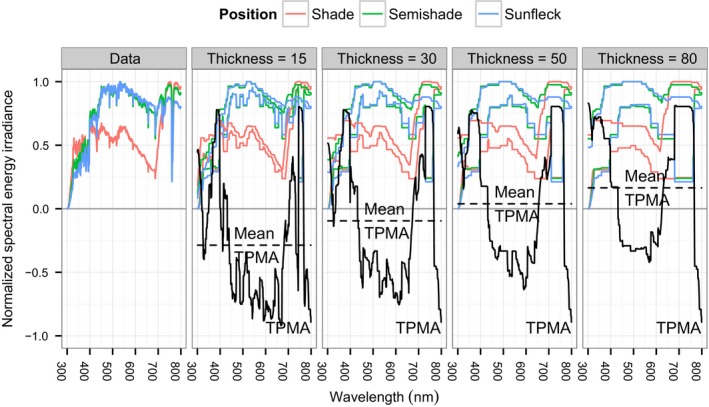
Normalized spectral energy irradiance, areas are formed by the thick pen transforms for selected thickness parameters, trivariate thick pen measure of association (TPMA) lines indicate the extent of overlap (solid black) for the three positions, and mean trivariate TPMA (dashed black). Data collected on the 22 May 2015 around solar noon in the BetulaOld stand

To quantify cross‐dependence between two or more measurements of spectral irradiance, we calculate the thick pen measure of association of Fryzlewicz and Oh (2011), TPMA, which reflects the overlap between the areas formed by their respective TPTs (see Figure 2 for an example with three measurements). Let ${\tilde{X}}^{\left(1\right)}={\left\{{\tilde{X}}_{\lambda }^{\left(1\right)}\right\}}_{\lambda \in [\lambda_{\min},\lambda_{\max}]}$ and ${\tilde{X}}^{\left(2\right)}={\left\{{\tilde{X}}_{\lambda }^{\left(2\right)}\right\}}_{\lambda \in [\lambda_{\min},\lambda_{\max}]}$ denote two normalized sequences, where the normalization is done via

(3) $${\tilde{X}}_{\lambda }=\frac{X_{\lambda }-\min_{\lambda_{\min}\leq l\leq \lambda_{\max}}X_{l}}{\max_{\lambda_{\min}\leq l\leq \lambda_{\max}}X_{l}-\min_{\lambda_{\min}\leq l\leq \lambda_{\max}}X_{l}}.$$

The normalization guarantees that the two records are on the same scale (Dengel et al., 2015; Fryzlewicz & Oh, 2011), which is needed when trying to measure the overlap between the areas formed by their respective TPTs. Since the minimum value for the measurement of the spectral irradiance is 0, we essentially divide the values of the measurement by the maximum value, obtaining a unit‐free sequence with values between 0 and 1. By comparing normalized measurements of spectral irradiance, we therefore compare their shapes. Let ${\text{TP}}_{T}\left({\tilde{X}}^{\left(1\right)}\right)$ and ${\text{TP}}_{T}\left({\tilde{X}}^{\left(2\right)}\right)$ be their corresponding TPTs for a given set of *n* thickness parameters $T=\{\tau_{1},\tau_{2},\ldots ,\tau_{n}\}$. The TPMA between them, for all λs and all $\tau_{i}$s, is defined as

(4) $$\rho_{\lambda }^{\tau_{i}}\left({\tilde{X}}^{\left(1\right)},{\tilde{X}}^{\left(2\right)}\right)=\frac{\min\left(U_{\lambda }^{\tau_{i}}\left({\tilde{X}}^{\left(1\right)}\right),U_{\lambda }^{\tau_{i}}\left({\tilde{X}}^{\left(2\right)}\right)\right)-\max\left(L_{\lambda }^{\tau_{i}}\left({\tilde{X}}^{\left(1\right)}\right),L_{\lambda }^{\tau_{i}}\left({\tilde{X}}^{\left(2\right)}\right)\right)}{\max\left(U_{\lambda }^{\tau_{i}}\left({\tilde{X}}^{\left(1\right)}\right),U_{\lambda }^{\tau_{i}}\left({\tilde{X}}^{\left(2\right)}\right)\right)-\min\left(L_{\lambda }^{\tau_{i}}\left({\tilde{X}}^{\left(1\right)}\right),L_{\lambda }^{\tau_{i}}\left({\tilde{X}}^{\left(2\right)}\right)\right)},$$

which is bounded by −1 from below and 1 from above, $\rho_{\lambda }^{\tau_{i}}\left({\tilde{X}}^{\left(1\right)},{\tilde{X}}^{\left(2\right)}\right)\in \left(-1,1\right]$ (it does not attain the lower value of −1). If the intervals $\left[L_{\lambda }^{\tau_{i}}\left({\tilde{X}}^{\left(1\right)}\right),U_{\lambda }^{\tau_{i}}\left({\tilde{X}}^{\left(1\right)}\right)\right]$ and $\left[L_{\lambda }^{\tau_{i}}\left({\tilde{X}}^{\left(2\right)}\right),U_{\lambda }^{\tau_{i}}\left({\tilde{X}}^{\left(2\right)}\right)\right]$ overlap, then the TPMA is positive and its value gives the ratio of the intersection to the union. If the intervals do not overlap, the TPMA is negative and its (absolute) value gives the ratio of the gap to (the shortest interval containing) the union. For example, focussing on Figure 2, and considering the thickness of 80 nm (right‐most panel) and the wavelength of 500 nm, the value of the bivariate TPMA for spectral irradiance from semi‐shade (green) and sunfleck (blue) is about 1 due to almost complete overlap. On the other hand, the bivariate TPMA for spectral irradiance from semi‐shade (green) and shade (red) is about −0.15/0.5 = −0.3: The negative sign indicates that the intervals marked by the respective pens at λ=500 nm are disjoint, the denominator of 0.5 is roughly the length of the union containing the semi‐shade and shade intervals, and 0.15 is approximately the length of the gap between those two intervals. The bivariate TPMA between shade and sunfleck would also be about −0.3.

Thick pen measure of association, just like TPT, is localized in wavelength (subscript λ) and scale (superscript $\tau_{i}$). If we average all its values over the wavelengths, ${\overset{¯}{\rho }}^{\tau_{i}}\left({\tilde{X}}^{\left(1\right)},{\tilde{X}}^{\left(2\right)}\right)=\frac{1}{\#\lambda }\sum_{\lambda }\rho_{\lambda }^{\tau_{i}}\left({\tilde{X}}^{\left(1\right)},{\tilde{X}}^{\left(2\right)}\right)$, then we will obtain an one‐number summary of the cross‐dependence between ${\tilde{X}}^{\left(1\right)}$ and ${\tilde{X}}^{\left(2\right)}$ for a given thickness. As already mentioned, Fryzlewicz and Oh (2011) provide a definition of the TPMA for the multivariate case, which boils down to modifying 4 accordingly. Namely, given *K* (normalized) measurements of spectral irradiance ${\tilde{X}}^{\left(1\right)}={\left\{{\tilde{X}}_{\lambda }^{\left(1\right)}\right\}}_{\lambda \in [\lambda_{\min},\lambda_{\max}]}$, ${\tilde{X}}^{\left(2\right)}={\left\{{\tilde{X}}_{\lambda }^{\left(2\right)}\right\}}_{\lambda \in [\lambda_{\min},\lambda_{\max}]}$, …, ${\tilde{X}}^{\left(K\right)}={\left\{{\tilde{X}}_{\lambda }^{\left(K\right)}\right\}}_{\lambda \in [\lambda_{\min},\lambda_{\max}]}$, the TPMA between them is

(5) $$\rho_{\lambda }^{\tau_{i}}\left({\tilde{X}}^{\left(1\right)},\ldots ,{\tilde{X}}^{\left(K\right)}\right)=\frac{\min_{k}\left(U_{\lambda }^{\tau_{i}}\left({\tilde{X}}^{\left(k\right)}\right)\right)-\max_{k}\left(L_{\lambda }^{\tau_{i}}\left({\tilde{X}}^{\left(k\right)}\right)\right)}{\max_{k}\left(U_{\lambda }^{\tau_{i}}\left({\tilde{X}}^{\left(k\right)}\right)\right)-\min_{k}\left(L_{\lambda }^{\tau_{i}}\left({\tilde{X}}^{\left(k\right)}\right)\right)},$$

for all values of λ and $\tau_{i}$. The properties of this multivariate measure are the same as those of its bivariate counterpart. The values of $\rho_{\lambda }^{\tau_{i}}\left({\tilde{X}}^{\left(1\right)},{\tilde{X}}^{\left(2\right)},{\tilde{X}}^{\left(3\right)}\right)$ for the example shown in Figure 2 are plotted as a black line and can also be found in the top‐left panel of Figure 8. The big hump in the TPMA around the wavelength of 700 nm, which is also present in the other plots of TPMA, is the effect of the concurrent drop in the spectral irradiance of all three measurements at approximately 762 nm, corresponding to absorption in the A‐band of oxygen in the atmosphere (Wark & Mercer, 1965). For a given $\tau_{i}$, the mean TPMA ${\overset{¯}{\rho }}^{\tau_{i}}\left({\tilde{X}}^{\left(1\right)},{\tilde{X}}^{\left(2\right)},{\tilde{X}}^{\left(3\right)}\right)$ is shown as a horizontal dashed line in Figure 2 and as a point in the middle panel of Figure 9.

## 3 RESULTS

**Figure 3:**
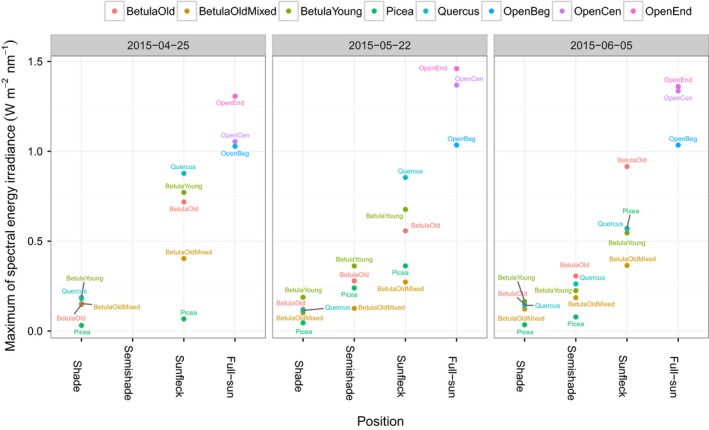
Maximum value of spectral energy irradiance, $\max_{\lambda_{\min}\leq l\leq \lambda_{\max}}X_{l}$, for all measurements

We begin with the simplest descriptive statistic of the data, namely, the maximum value of the spectral irradiance at any given wavelength within the range 300–800 nm (Figure 3), which will be used to normalize each *X*. As would be expected, the maximum spectral irradiance (W m^−2^ nm^−1^) is smallest in the shade position with the values of up to 0.25, up to 0.50 in semi‐shade, and up to 1.00 in sunflecks. The values corresponding to the position full‐sun fall between 1 and 1.5 (W m^−2^ nm^−1^). The variability within each position among the stands (their vertical spread) follows the ranking from low to high for shade, semi‐shade, and sunfleck.

Next, we apply the thick pen transform (Rcode can be found in Supporting Information Appendix S2) to the normalized spectral irradiance with thickness parameters (in nm) ∈{15,20,25,30,40,50,60,70,80}; these parameters yield a representative cross‐section of scales ranging from small to large.

### 3.1 Cross‐dependence of the spectra with respect to date, stand, and position

Throughout this section, we work with the thick pen transform of the normalized data, $\tilde{X}$. To facilitate comparisons, the vertical range of the *y*‐axis in all graphs of TPMA is 2 and in all graphs of mean TPMA it is 1.5.

**Figure 4:**
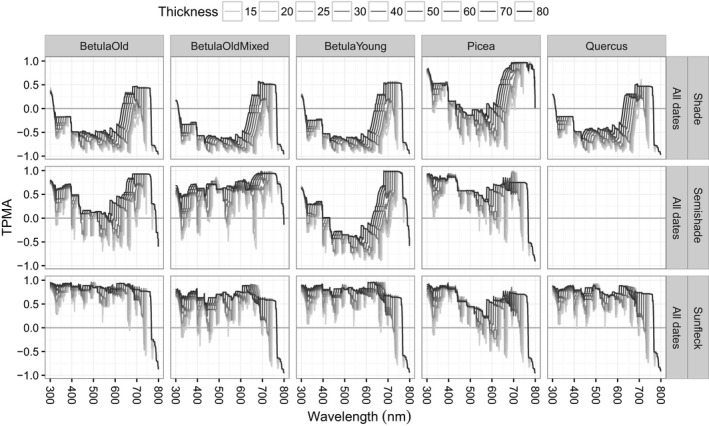
Thick pen measure of association for spectral irradiance from all dates (25 April 2015, 22 May 2015, 05 June 2015) for a given position and for a given stand. Only June data for semi‐shade in Quercus available

**Figure 5:**
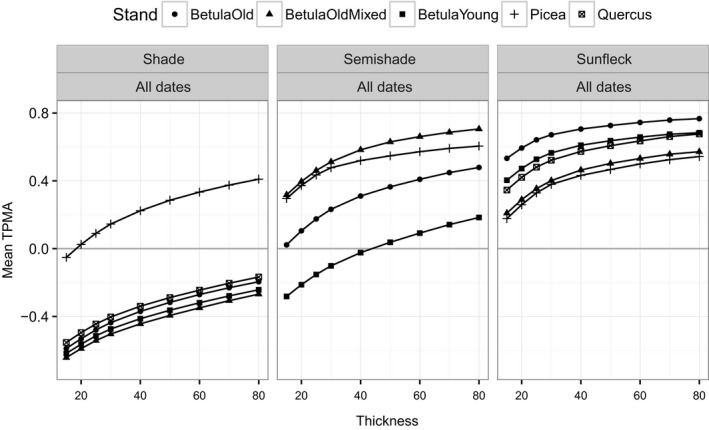
Thick pen measure of association for spectral irradiance from all dates (25 April 2015, 22 May 2015, 05 June 2015) averaged over wavelengths, for a given position and for a given stand

We begin with an overview of cross‐dependence of spectral irradiance w.r.t. date (Figures 4 for the TPMA and 5 for the mean TPMA), namely, for a given position and for a given stand, we compare the shapes of spectral irradiance recorded on different dates. The spectra from the shade position are the least similar, giving a negative mean TPMA for all stands except for Picea, at all thickness values (left panel of Figure 5). This was partially caused by the negative values of TPMA for wavelengths [315, 620] nm compared to other wavelengths (top panels of Figure 4) and may reflect the higher PAI in Picea compared with the other stands (Table 1). Spectra from the semi‐shade position come second in the ranking of similarity producing a positive mean TPMA except for BetulaYoung (middle panel of Figure 5). In this stand, April, May, June spectra are deemed similar in average terms (positive mean TPMA) only if sufficiently large thickness values are used, that is, if large‐scale features are considered. Spectra in the sunfleck position are the most similar (mean TPMA between 0.2 and 0.8, right panel of Figure 5). The association of spectra in the sunfleck position exhibits the smallest differences with respect to the different stands, although this would not have been the case if Picea had been ignored in shade position, because all the other stands in the shade position yield similar (and negative) mean TPMA. The semi‐shade position produces large variation in mean TPMA among stands. The rate of change in mean TPMA w.r.t. thickness (the steepness of curves in Figure 5) depends on stand identity and position. For example, in BetulaOld, the mean TPMA changes least in the sunfleck and most in semi‐shade. Hence, date has the weakest effect on the spectral irradiance in the sunfleck position (spectra remain similar despite pooling across April, May, June), and this is true for all the stands at all thickness values. However, date produces spectral differences in the shade position in non‐Picea stands, at all thickness values. The effect of date in the semi‐shade position varies from weak (similarity of April, May, and June spectra from BetulaOldMixed is high) to strong (similarity of April, May, and June spectra in BetulaYoung is low).

**Figure 6:**
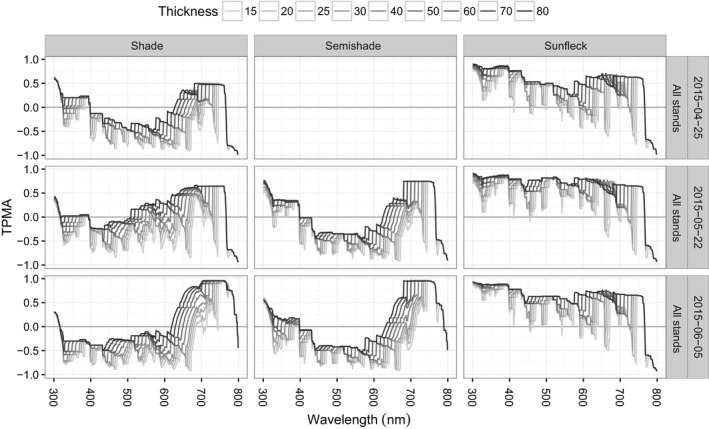
Thick pen measure of association for spectral irradiance from all stands (BetulaOld, BetulaOldMixed, BetulaYoung, Picea, Quercus) for a given position and for a given date. No data for semi‐shade in April

**Figure 7:**
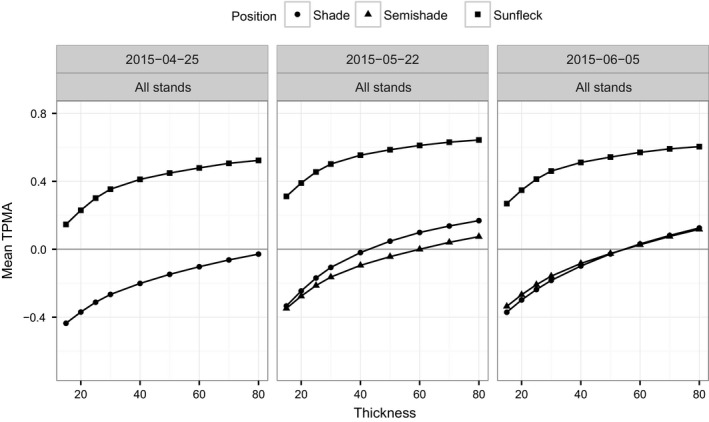
Thick pen measure of association for spectral irradiance from all stands (BetulaOld, BetulaOldMixed, BetulaYoung, Picea, Quercus) averaged over wavelengths, for a given position and for a given date

When we pool all the stands together and measure the cross‐dependence of spectral irradiance for a given position and date (Figures 6 for the TPMA and 7 for the mean TPMA), only the sunfleck position has spectra that overlap with each other (mean TPMA between 0.2 and 0.6, Figure 7), revealing fairly small changes w.r.t. date (see also the last column of Figure 6). The other positions yield negative values of the mean TPMA in April for the whole range of thickness values tested (left panel of Figure 7) and negative values for all but large thicknesses in May and June (middle and right panels of Figure 7). In the semi‐shade position (middle panels of Figure 6), the spectra from all the stands seem to particularly diverge for wavelengths between 450 and 580 nm on both dates and for all thickness values. In the shade position (left panels of Figure 6), the wavelength interval where the spectra of all stands are dissimilar (negative TPMA) changes with date from [400, 620] nm in April, through [400, 500] nm in May, to [320, 620] nm in June, for all thickness values. Therefore, stand has some influence on the shape of spectral irradiance collected in the sunfleck position (stronger than date because mean TPMAs w.r.t. stand are smaller than those w.r.t. date) and a fairly strong influence on the spectra obtained in the shade and semi‐shade (because in those positions spectra are dissimilar w.r.t. stand leading to small or negative mean TPMA).

**Figure 8:**
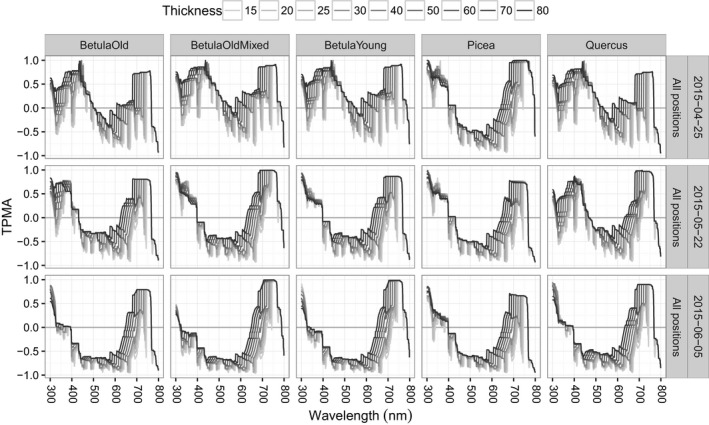
Thick pen measure of association for spectral irradiance from all positions (shade, semi‐shade, and sunfleck) for a given stand and for a given date

**Figure 9:**
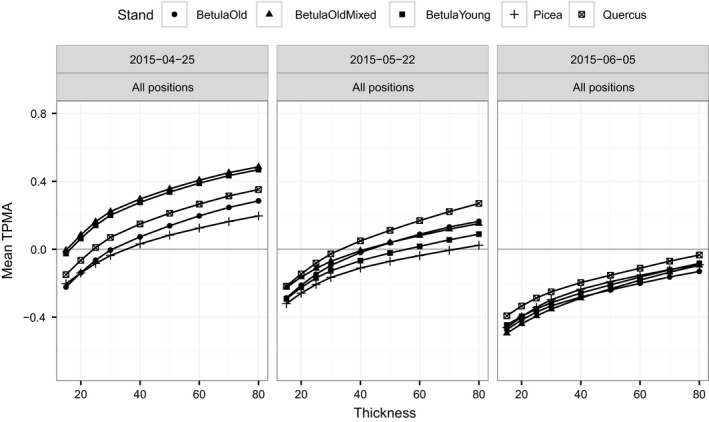
Thick pen measure of association for spectral irradiance from all positions (shade, semi‐shade, and sunfleck) averaged over wavelengths, for a given stand and for a given date

To assess the influence of position on the cross‐dependence of spectra, we combine data for a given date and given stand (Figures 8 for the TPMA and 9 for the mean TPMA). In average terms, the impact of position is stronger than that of date and stand, as the mean TPMA never exceeds 0.5 (Figure 9). The largest mean TPMA values are from April, which is mainly due to the spectral similarity over wavelengths of up to 500 nm (top panels of Figure 8) except for in Picea. Cross‐dependence decreases with date and so do the differences in cross‐dependence for different stands (smaller vertical spread), culminating in negative mean TPMA for all thickness values in June.

To complement multivariate TPMA, we also perform bivariate comparisons of spectra (results not shown) for the following pairs: shade and semi‐shade, semi‐shade and sunfleck, and shade and sunfleck. Because the mean trivariate TPMA behaves similarly to the mean bivariate TPMA for the shade and sunfleck positions, we can conclude that the latter pair largely controls the overlap between the three areas marked by pens of different thicknesses. The impact of stand identity on the cross‐dependence between the spectra for shade and sunfleck is the smallest (a vertical spread up to 0.3), and it appears to be slightly stronger for shade and semi‐shade (a vertical spread up to 0.5) and particularly strong (a vertical spread in the mean TPMA for all $\tau_{i}$s of up to 1 for the earlier date) for semi‐shade and sunfleck positions. The bivariate TPMA (like the trivariate TPMA) w.r.t. position decreases with date. Overall, spectra for semi‐shade and sunfleck positions seem to co‐depend more than those for shade and semi‐shade, which in turn are more correlated than those for shade and sunfleck positions. Interestingly, the impact of position in the BetulaOldMixed stand (second column of Figure 8) is quite similar to that on BetulaOld and BetulaYoung stands (first and third columns of Figure 8), leading to a negative TPMA caused by dissimilarity between shade and sunfleck positions. However, in the semi‐shade position, when all the dates were pooled, the influence of date on BetulaOldMixed (second column, middle row of Figure 4) was quite different (much weaker because the TPMA is large) from that on BetulaOld and BetulaOldYoung (first and third columns, middle row of Figure 4).

To complete these comparisons of cross‐dependence among spectra, we compare spectra recorded in the open (position full‐sun) with those recorded at each of the three positions (sunfleck, semi‐shade, and shade) under the forest canopy (Figures A.1–A.6 can be found in Supporting Information Appendix S1). We start with sunfleck and full‐sun (Figures A.1 for the TPMA and A.2 for the mean TPMA). All values of the mean TPMA are positive, ranging between 0.2 and 0.8, indicating a relatively high degree of similarity. Compared to the other stands, the mean TPMA values are lower in Picea in April and higher in Quercus in May, while in June, the results w.r.t. stand are quite similar. There appears to be a downward trend w.r.t. date. When it comes to the spectra measured in semi‐shade and full‐sun positions (Figures A.3 for the TPMA and A.4 for the mean TPMA), their degree of similarity is determined largely by stand identity. In May, spectra from semi‐shade and full‐sun positions are fairly similar in BetulaOld and Picea at all thickness values, but are quite different in BetulaOldMixed at most thickness values. In June, Picea has the greatest similarity between semi‐shade and full‐sun: This might reflect differences in stand structure and PAI (Table 1) affecting the size of sunflecks and properties of semi‐shade due to clumping, or optical differences between needle‐leaved and broad‐leaved canopies. The least similar stands in June are BetulaOldMixed and Betula Young, with negative mean TPMA at all thickness values. The similarity decreases with time of year through the spring. Finally, the spectra measured in shade and full‐sun positions (Figures A.5 for the TPMA and A.6 for the mean TPMA) correlate positively only over fairly large $\tau_{i}$s, for all stands in April apart from Picea, whose mean TPMA is between −0.3 and 0. The spectra in the shade and sunfleck become progressively less similar as we move onto May and June, culminating in a negative mean TPMA in June (values between −0.6 and −0.1, right panel of Figure A.6). To sum up, spectra recorded in sunflecks resemble those recorded in full‐sun at all thickness values, with small variations w.r.t. stand and date. The similarity of spectra from semi‐shade and full‐sun positions varies greatly and is highly dependent on stand identity. Spectra from the shade are the least similar to those in full‐sun, with some variation w.r.t. stand and date.

At the main factor level, position had the strongest impact on the spectral composition followed by stand identity and then date. Considering interactions, the following combinations caused the spectra to have different shapes: date in shade; stand in shade; stand in semi‐shade, and position in June. On average, the degree of cross‐dependence increased with increasing thickness, but the rates of change differed, as negativity/positivity was sometimes dependent on the thickness considered.

## 4 DISCUSSION

Of the various factors examined, position (understorey position with respect to sunlight) had the strongest influence on spectral irradiance: That is, spectra from sunflecks, semi‐shade, and shade positions compared by TPMA were least similar overall and became ever more dissimilar as the spring progressed (Figure 9). In the past, this distinction among positions has often been overlooked or impossible to measure due to technical restrictions (Vezina & Boulter, 1966 and references therein, Leuchner et al., 2011; Smith & Berry, 2013; Pearcy & Way, 2012), and studies have primarily focussed on describing diffuse shade in understoreys (e.g., Freyman, 1968).

The vertical spread among the mean TPMA values of stands when comparing positions was smaller across successive dates, meaning that the influence of stand identity on the differences among positions became smaller through the spring. Despite the later leaf‐out in the Quercus stand compared to the *Betula* stands, the composition of their spectral irradiance converged during spring because leaf flush of the *Q. robur* canopy subsequently proceeded quickly and caught up with that of *Betula* (Table 1).

When spectra from all stands were compared using the mean TPMA, we also saw that of the three positions, sunfleck spectra were the least influenced by stand identity, although stand identity's influence was nevertheless greater than that of date (Figure 7). The result for shade was similar to semi‐shade, and both positions were dissimilar with respect to stand at all but highest TPT thicknesses.

The extent to which spectral composition changed with date differed between the sunfleck, semi‐shade and shade positions. Cross‐correlations of mean TPMA revealed the strongest effect of date in shade (Figure 5), possibly due to leaf‐out during the course of the experiment in all stands apart from Picea (Table 1). Whereas the effect of date was weakest in sunflecks, this would be consistent with the majority of radiation in sunflecks being direct (Smith & Berry, 2013). The results of studies that report high seasonal variation in irradiance due to leaf‐out and solar elevation (Hutchison & Matt, 1977), as well as differences in spectral composition between conifer and broad‐leaved canopies (Federer & Tanner, 1966; Vezina & Boulter, 1966), are cogent with this result.

These comparisons enable us to build up a hierarchy of effects on spectral composition (position, stand identity, date, etc.). The time‐and‐canopy‐identity‐dependent differences in the understorey light environment we have identified are likely to be of ecological importance for the forest ecosystem (Messier & Belleeur, 1988): for example, through photoreceptor‐mediated plant responses (Casal, 2013; Mazza & Ballaré, 2015). But, looking beyond forest understoreys, the TPT can be applied in other fields where simultaneous spectral analysis is required. In the future, more frequent utilization of array spectroradiometers for measurements as well as modeling of spectral irradiance (Lindfors & Arola, 2008) will likely result in a greater need to analyze large datasets of spectral data.

Alternative approaches to TPT that could potentially be used for comparing curves pertain to the frameworks of functional data analysis (Ramsay & Silverman, 2005) and wavelet analysis (Percival & Walden, 2000). In functional data analysis, the curves are required to be smooth at the outset; hence, comparison of signals like those presented in the current paper without smoothing is not possible and inherently this approach lacks the multi‐scale perspective. Although wavelet analysis is not restricted to smooth signals and offers the multi‐scale dimension, the data have to be sampled on an equispaced grid, which would require interpolation, and if the discrete variant of the wavelet transform is used, the scales can only be dyadic (2 raised to an integer power). Both approaches admit only bivariate comparisons based on cross‐correlation operators and are perhaps not as computationally accessible as TPT, whose building blocks are running minima and maxima.

## CONFLICT OF INTEREST

None declared.

## AUTHOR CONTRIBUTIONS

SMH, AJ, AG, and TMR conceived the ideas for the manuscript. SMH and TMR designed the field methodology. SMH collected the data. AJ, AG, and TMR analyzed the data. SMH and TMR led the writing of the manuscript. All authors contributed critically to the drafts and gave final approval for publication.

## DATA ACCESSIBILITY

Measurements of spectral irradiance: Zenodo https://doi.org/10.5281/zenodo.1246597.
