# Supplementary material for: Assessing scale‐wise similarity of curves with a thick pen: As illustrated through comparisons of spectral irradiance
Source: Ecol Evol. 2018 Sep 12;8(20):10206–18. doi: 10.1002/ece3.4496 (PMC6206219; doi:10.1002/ece3.4496)
Supplement: Supplementary file 1 [file ECE3-8-10206-s001.pdf]

Assessing scale-wise similarity of curves with a thick pen: as illustrated through comparisons of spectral irradiance

Saara M. Hartikainen[1], Agnieszka Jach[2], Aurea Grané[3], T. Matthew Robson[1,*]

[1] *University of Helsinki, Viikki Plant Science Centre, PB 65, Helsinki, Finland;* [2] *Department of Finance and Statistics, Hanken School of Economics, PB 479, Helsinki, Finland;* [3] *Department of Statistics, Universidad Carlos III de Madrid, C/ Madrid, 126, Getafe, Spain*

Appendix: Graphs

*Correspondence author. E-mail: matthew.robson@helsinki.fi

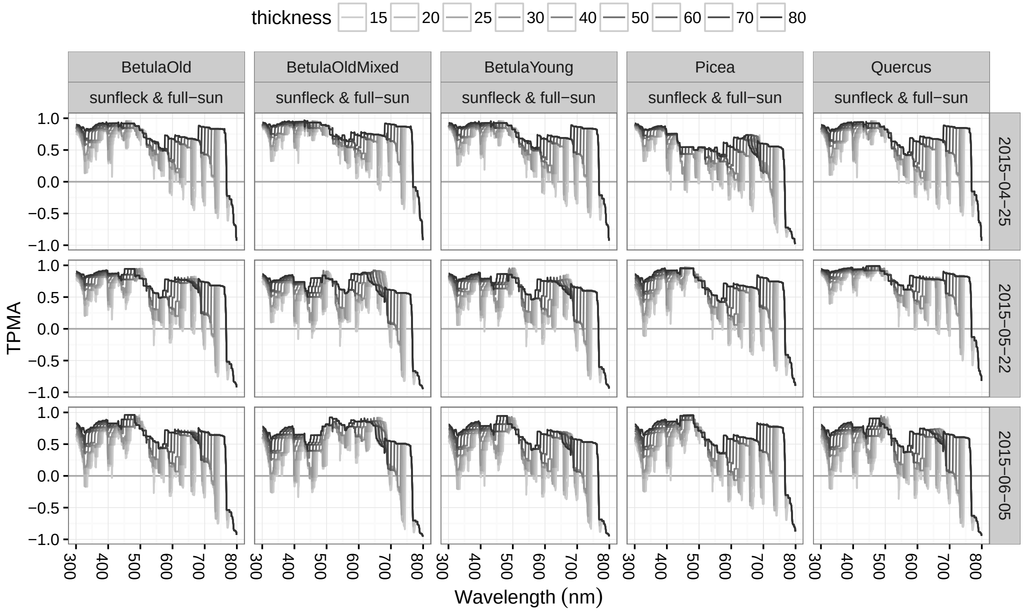

Figure A.1: TPMA for spectral irradiance from positions sunfleck and full-sun, for a given stand and for a given date.

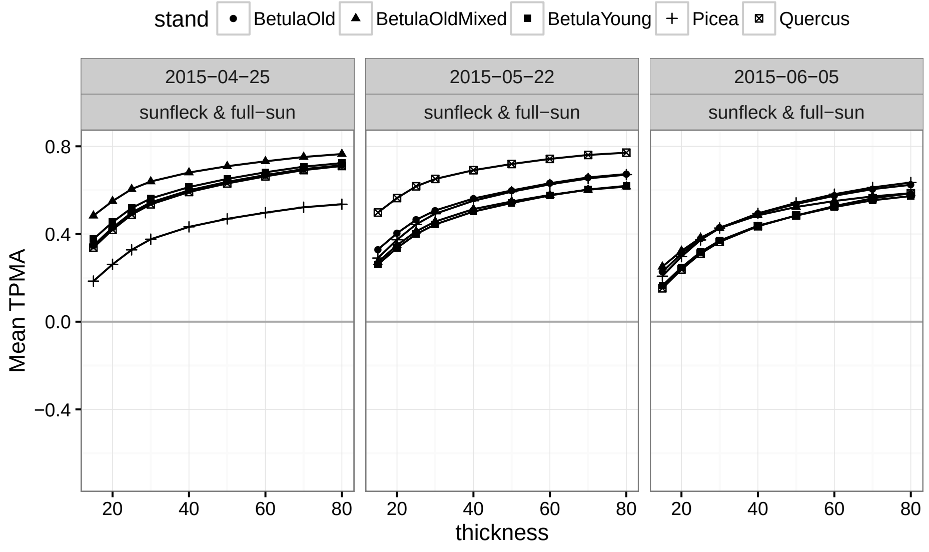

Figure A.2: TPMA for spectral irradiance from positions sunfleck and full-sun, averaged over wavelengths, for a given stand and for a given date.

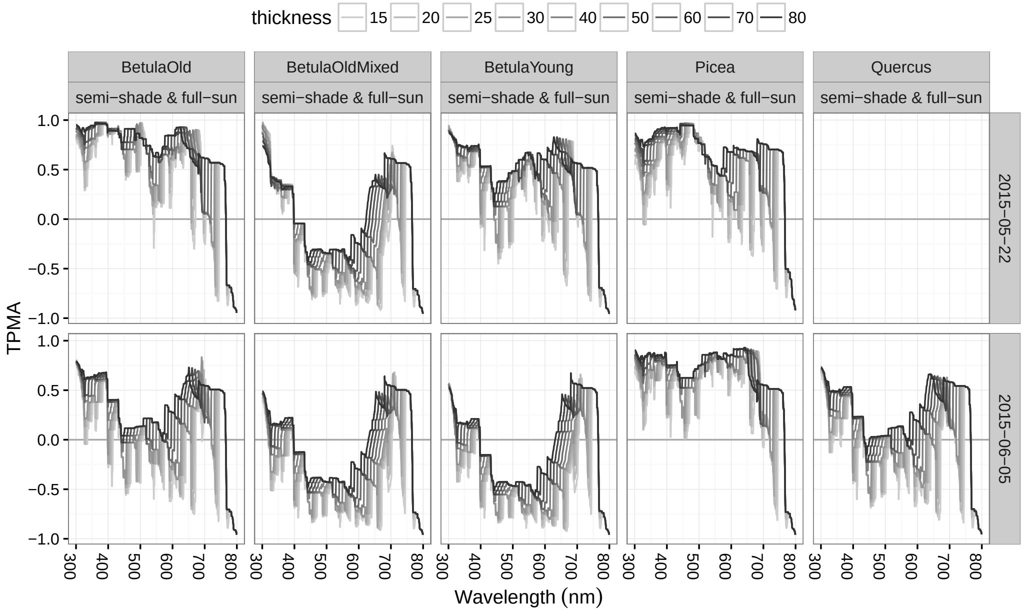

Figure A.3: TPMA for spectral irradiance from positions semi-shade and full-sun, for a given stand and for a given date. No data for semi-shade in Quercus.

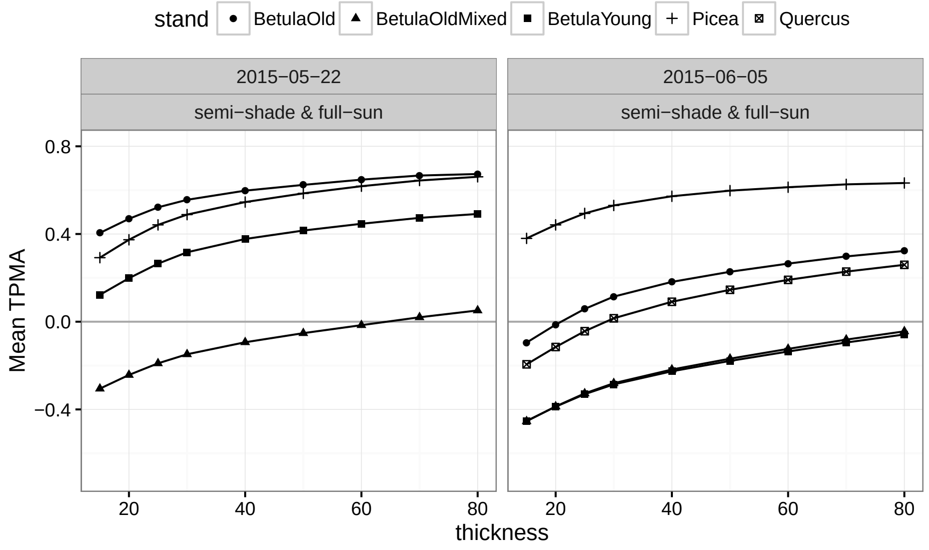

Figure A.4: TPMA for spectral irradiance from positions semi-shade and full-sun, averaged over wavelengths, for a given stand and for a given date.

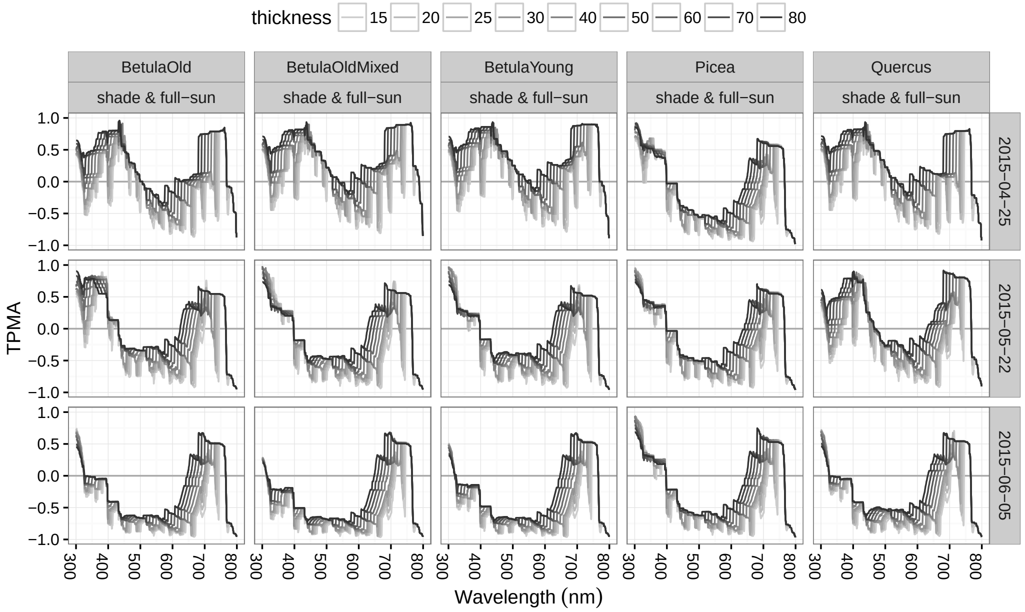

Figure A.5: TPMA for spectral irradiance from positions shade and full-sun, for a given stand and for a given date.

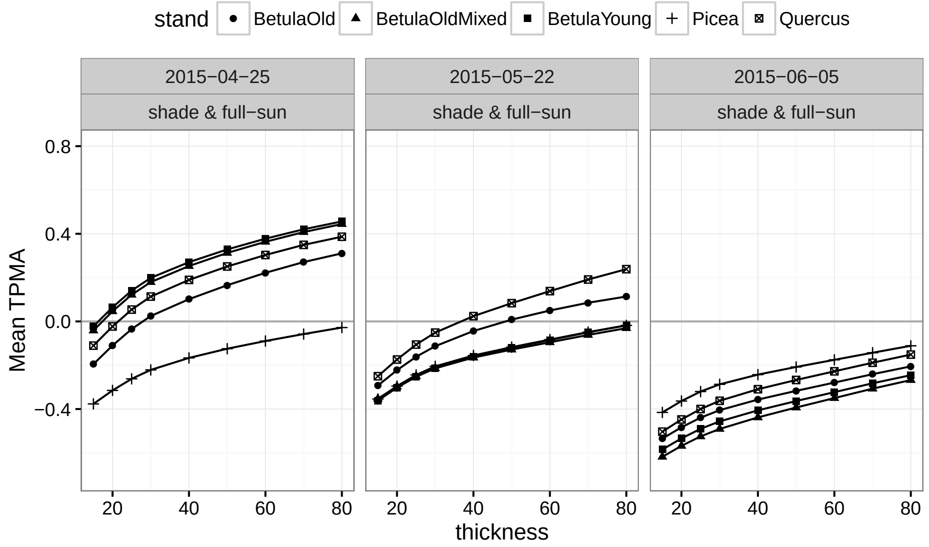

Figure A.6: TPMA for spectral irradiance from positions shade and full-sun, averaged over wavelengths, for a given stand and for a given date.
